# Mitochondrial p53 phosphorylation induces Bak-mediated and caspase-independent cell death

**DOI:** 10.18632/oncotarget.3780

**Published:** 2015-04-23

**Authors:** Jinjing Wang, Wenhao Guo, Hang Zhou, Na Luo, Chunlai Nie, Xinyu Zhao, Zhu Yuan, Xinyu Liu, Yuquan Wei

**Affiliations:** ^1^ Department of Abdominal Oncology, State Key Laboratory of Biotherapy and Cancer Center, West China Hospital, Sichuan University and Collaborative Innovation Center for Biotherapy, Chengdu 610041, P. R. China; ^2^ Department of Chemotherapy, Sichuan Cancer Hospital, Chengdu, Sichuan 610041, P. R. China; ^3^ Nankai University School of Medicine/Collaborative Innovation Center of Biotherapy, Tianjin 300071, P.R. China

**Keywords:** Akt, Bak, p53, caspase-independent

## Abstract

Chemoresistance in cancer has previously been attributed to gene mutations or deficiency. Caspase mutations or Bax deficiency can lead to resistance to cancer drugs. We recently demonstrated that Bak initiates a caspase/Bax-independent cell death pathway. We show that Plumbagin (PL) (5-hydroxy-2-methyl-1,4-napthoquinone), a medicinal plant-derived naphthoquinone that is known to have anti-tumor activity in a variety of models, induces caspase-independent cell death in HCT116 Bax knockout (KO) or MCF-7 Bax knockdown (KD) cells that express wild-type (WT) Bak. The re-expression of Bax in HCT116 Bax KO cells fails to enhance the PL-induced cell death. Additionally, Bak knockdown by shRNA efficiently attenuates PL-induced cell death. These results suggest that PL-induced cell death depends primarily on Bak, not Bax, in these cells. Further experimentation demonstrated that p53 Ser15 phosphorylation and mitochondrial translocation mediated Bak activation and subsequent cell death. Knockdown of p53 or a p53 Ser15 mutant significantly inhibited p53 mitochondrial translocation and cell death. Furthermore, we found that Akt mediated p53 phosphorylation and the subsequent mitochondrial accumulation. Taken together, our data elaborate the role of Bak in caspase/Bax-independent cell death and suggest that PL may be an effective agent for overcoming chemoresistance in cancer cells with dysfunctional caspases.

## INTRODUCTION

Cancer is a leading cause of death worldwide and accounted for 7.6 million deaths (approximately 13% of all deaths) in 2008. Deaths from cancer are projected to continue rising, with an estimated 13.1 million deaths projected to occur in 2030 [1, 2]. Therefore, it is necessary to study tumorigenesis mechanisms to find more effective treatment methods. However, both intrinsic and acquired drug resistance are serious problems in cancer treatment.

Recent evidence has suggested that the failure of drug-induced apoptosis may be an underlying cause of resistance in cancers. Recent studies have identified several key mediators of apoptosis that are mutated in chemoresistant cancer cells, such as p53 and Akt [3, 4]. The caspase family of cysteinyl-proteases plays key roles in the initiation and execution of programmed cell death, and mutations in these proteins have been speculated to be the leading cause of cancer therapy chemoresistance [5, 6]. Triggering the caspase independent death pathways has thus become an attractive alternative approach to eradicating tumor cells.

The mitochondria are central relaying stations for both caspase-dependent and caspase-independent death pathways [7, 8]. Mitochondria respond to multiple death stimuli, such as those of the pro-apoptotic Bcl-2 family of proteins, which includes Bax/Bak; these proteins induce mitochondrial membrane permeabilization and the release of apoptotic molecules such as Smac, AIF and endoG [9–11]. AIF and endoG start a death pathway that can execute apoptosis-like cell death in the absence of caspase [10, 12]. Bax can induce the release of AIF in caspase-independent cell death [13]. Bak can trigger the release of Cytochrome c (Cyt c) from mitochondria in HCT Bax KO cells [14]. However, the function of Bak, excluding the effect of Bax, in caspase-independent cell death is still unclear.

Our previous study demonstrated that Akt could mediate Bax activation through p53 mitochondrial accumulation [9]. In the present study, we found that Akt could induce p53 phosphorylation and mitochondrial translocation. Activated p53 triggers Bak-dependent and Bax/caspase-independent cell death. These results will help us better understand the function of Bcl-2 protein family members in apoptosis and cancer therapy. Furthermore, our experiments may provide a theoretical basis for overcoming chemoresistance in cancer cells.

## RESULTS

### PL induces caspase-independent cell death

**Figure 1:**
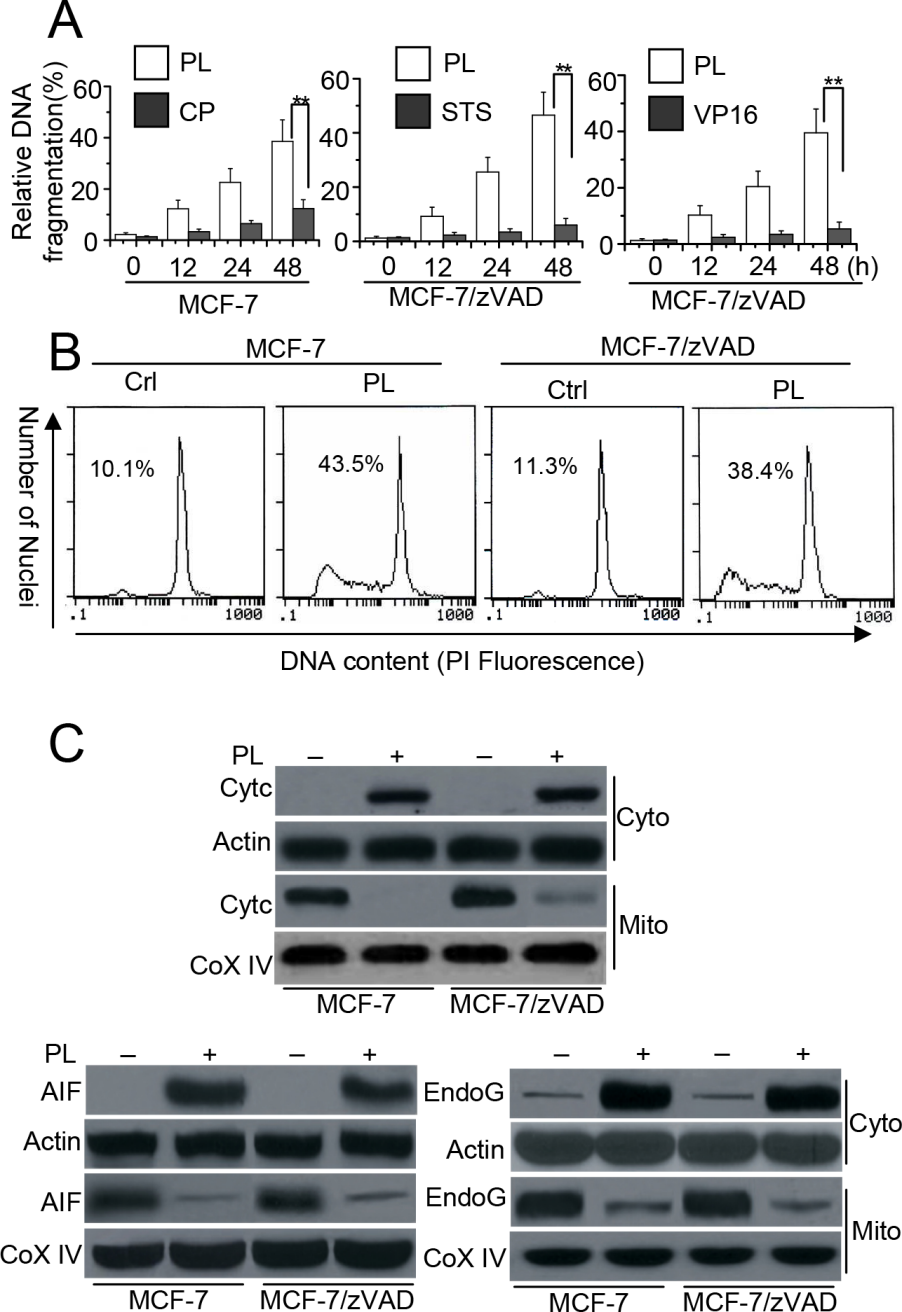
PL induces cell death in cancer cells independent of caspase **A.** Analysis of cell apoptosis treated with PL or chemotherapeutic drug. MCF-7 cells were treated with PL (3 μM) or cisplatin (CP) (10 μM) for different periods of time. Meanwhile, MCF-7/zVAD(MCF-7 cells were pretreated with 20 μM zVAD) cells were pretreated with zVAD (20 μM) for 1 h and then treated with PL (10 μM), or staurosporine (STS) (2 μM), etoposide (VP16) (100 μM) at indicated time. Cell death was quantitatively detected by a cell death ELISA kit as described in Materials and methods. Graphs showing results of quantitative analyses (*n* = 3, mean ± S.D. **, *P* < 0.01). **B.** MCF-7 cells or MCF-7/zVAD were exposed to 3 μM (MCF-7) or 10 μM PL (MCF-7/zVAD) for 48 h, and then collected for PI staining. Sub-G1 cells (apoptotic cells), respectively, as assessed by flow cytometry. **C.** Cells were treated with 10 μM PL for 48 h, and then subjected to subcellular fractionation. The cytosolic and mitochondrial fractions were immunoblotted for Western detection. The used concentrations of agents are described in **B.** β-Actin and Cox IV was used as a protein loading control. Data are representative of at least three independent experiments.

We first determined the apoptotic effect of PL in MCF-7 (caspase-3 deficient) cells. We treated the cells with PL at the indicated concentrations, and apoptosis was assessed by a DNA fragmentation ELISA assay. As depicted in Figure 1A, PL efficiently induced cell death in MCF-7 cells. CP treatment, however, had no effect on cell death. We then used a pan caspase inhibitor in MCF-7 cells called MCF-7/zVAD. Even when the caspase activity was inhibited by zVAD, PL treatment was still sufficient to induce cell death. However, STS or VP16 treatment had no effect on cell death (Figure 1A). Flow cytometry analysis with PI staining revealed that PL could induce cell death in MCF-7 and MCF-7/zVAD cells (Figure 1B). Further experiments demonstrated that PL effectively induces Cyt c, AIF and endoG release from the mitochondria to the cytosol (Figure 1C). AIF condenses chromatin and endoG cleaves chromatin DNA into nucleosomal fragments independently of caspases [12, 15], leading to cell death. Our data also demonstrate that either AIF or endoG siRNA only partially attenuated cell death but that AIF and endoG double siRNA efficiently inhibited cell death by PL treatment (Supplementary Figure 1).

### Bak activation is necessary for PL-induced caspase-independent cell death

**Figure 2:**
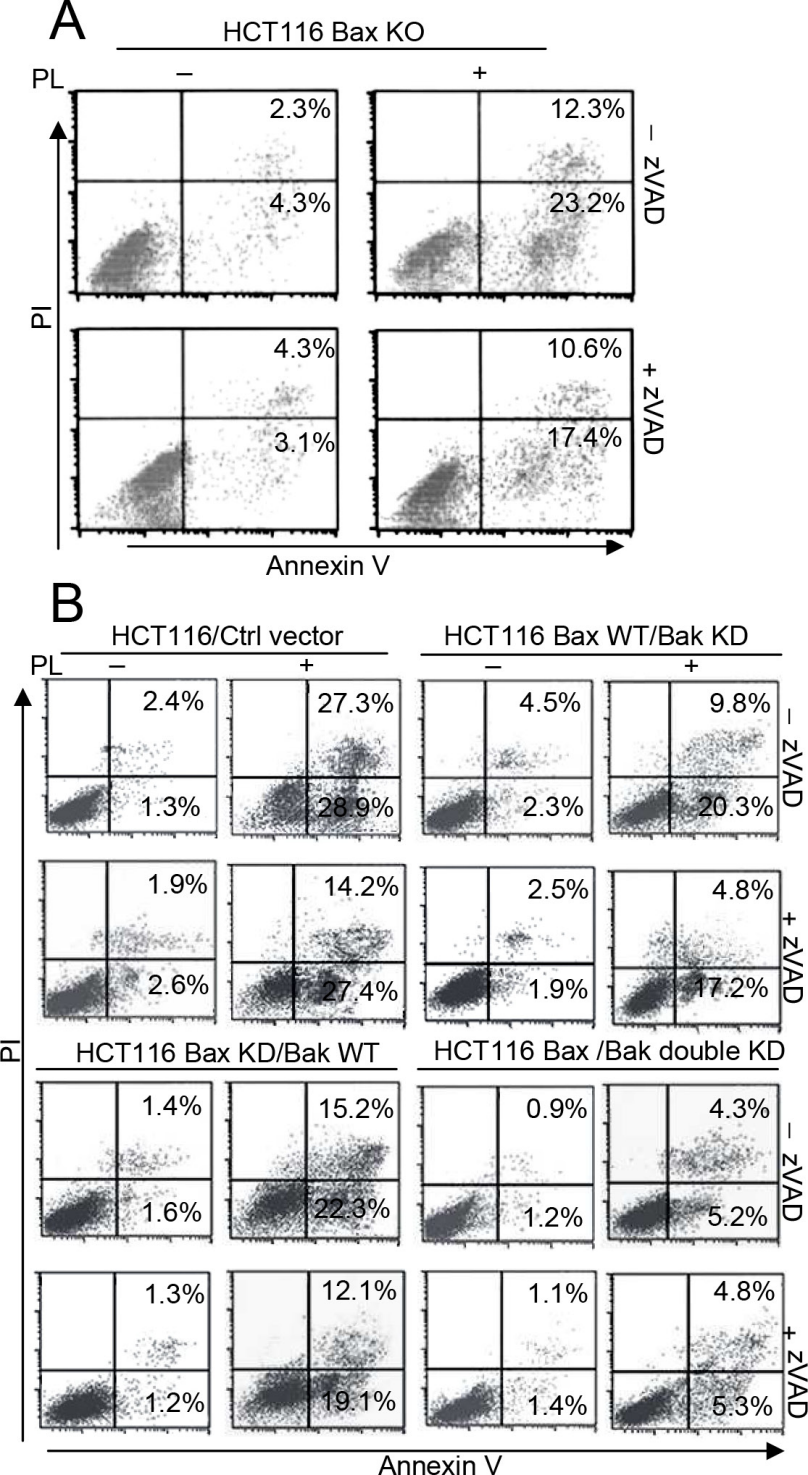
Bak not Bax is necessary for PL-induced cell death **A.** HCT116 Bax KO cells were pretreated with or without 20 μM zVAD for 1 h and with 10 μM PL for 48 h. Cells were collected for Annexin V/PI staining to detect cell apoptosis. **B.** HCT116 cells were transfected with ctrl vector, Bax, Bak shRNA, or double shRNA for 48 h to obtain the described different HCT116 cells. Cells were pretreated with or without 20 μM zVAD for 1 h and then treated with PL for 48 h and treated cells were collected for Annexin V/PI staining to detect cell apoptosis. Representative results of three experiments with consistent results are shown.

We also found that PL induced apoptosis in HCT116 Bax KO cells. Moreover, the inhibition of caspase activity by zVAD only partially prevented cell death in PL-treated HCT116 Bax KO cells (Figure 2A). We treated HCT116 Bax KO with an anti-Fas antibody and cycloheximide (CHX), as previously described [16], and found that the combined treatment of the anti-Fas cross-linking antibody and CHX resulted in caspase-3 cleavage and cell death (Supplementary Figure 2A). Caspase-3 activation and cell death, however, were impeded in the presence of zVAD (Supplementary Figure 2A and 2B), as previously reported [16]. Similarly, using immunofluorescent staining, we found that PL induced the release of Cyt c, AIF and endoG in HCT116 Bax KO cells (Supplementary Figure 3). We also examined the effect of PL on HCT116 cells. We found that PL triggers casapse-3 activation in HCT116 or HCT116 Bax KO cells but that zVAD treatment inhibited caspase-3 cleavage (Supplementary Figure 2C). However, caspase inactivation by zVAD could not efficiently decrease the PL-induced cell death in HCT116 or HCT116 Bax KO cells (Supplementary Figure 2D and 2E). These results indicate that PL treatment can induce caspase-dependent and caspase-independent cell death. Moreover, caspase-independent cell death is necessary for PL-induced cell death.

**Figure 3:**
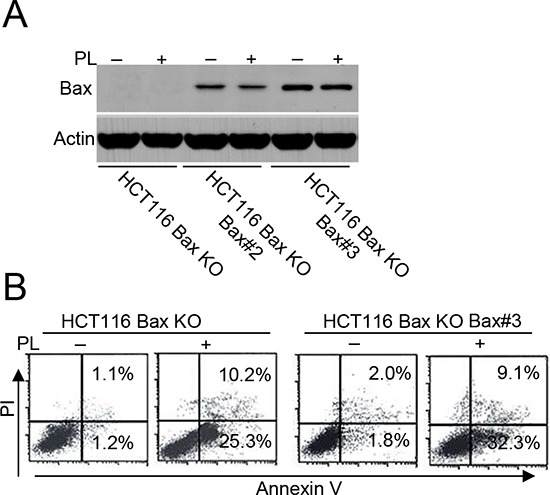
Bax restoration could not efficiently enhance cell death **A.** HCT 116 Bax KO cells were transfected with pCDNA-Bax construct and G418 selection to get stable Bax transfectants HCT 116 Bax KO Bax#2 and Bax#3. Cells were collected for Bax immunoblotted for Western detection. β-Actin was used as a protein loading control. **B.** HCT116 Bax KO and HCT 116 Bax KO Bax#3 cells were treated with PL for 48 h, and then treated cells were collected for Annexin V/PI staining to detect cell apoptosis. All data are representative of three independent experiments.

Our data demonstrate that PL can trigger cell death in HCT116 Bax KO cells. Because Bak contributes to Bax-independent cell death [14], we speculated that Bak could mediate PL-induced Bax/caspase-independent cell death. To compare the impact of Bak and Bax on cell death, we transfected Bax, Bak or both shRNA into HCT116 or MCF-7 cells to obtain different cell lines (Supplementary Figure 4A and 4B). We then detected cell death in the different HCT116 cells after PL treatment with or without the addition of zVAD treatment (Figure 2B). We found that Bak had a more important role in PL-induced cell death than did Bax (Figure 2B and Supplementary Figure 4C), although our data revealed that PL could induce Bax activation in MCF-7 cells (Supplementary Figure 4D). We then transfected a Bax vector into HCT116 Bax KO cells to obtain stable the Bax transfectant clones Bax#2 and Bax#3 (Figure 3A). We selected Bax#3 as a cell model because Bax expression was highest in this clone. We found that the restoration of Bax expression was not effective in increasing death following PL treatment (Figure 3B). These results confirm that cell death by PL does not depend on the loss of Bax and suggest that PL utilizes the Bak pathways to execute cell death.

**Figure 4:**
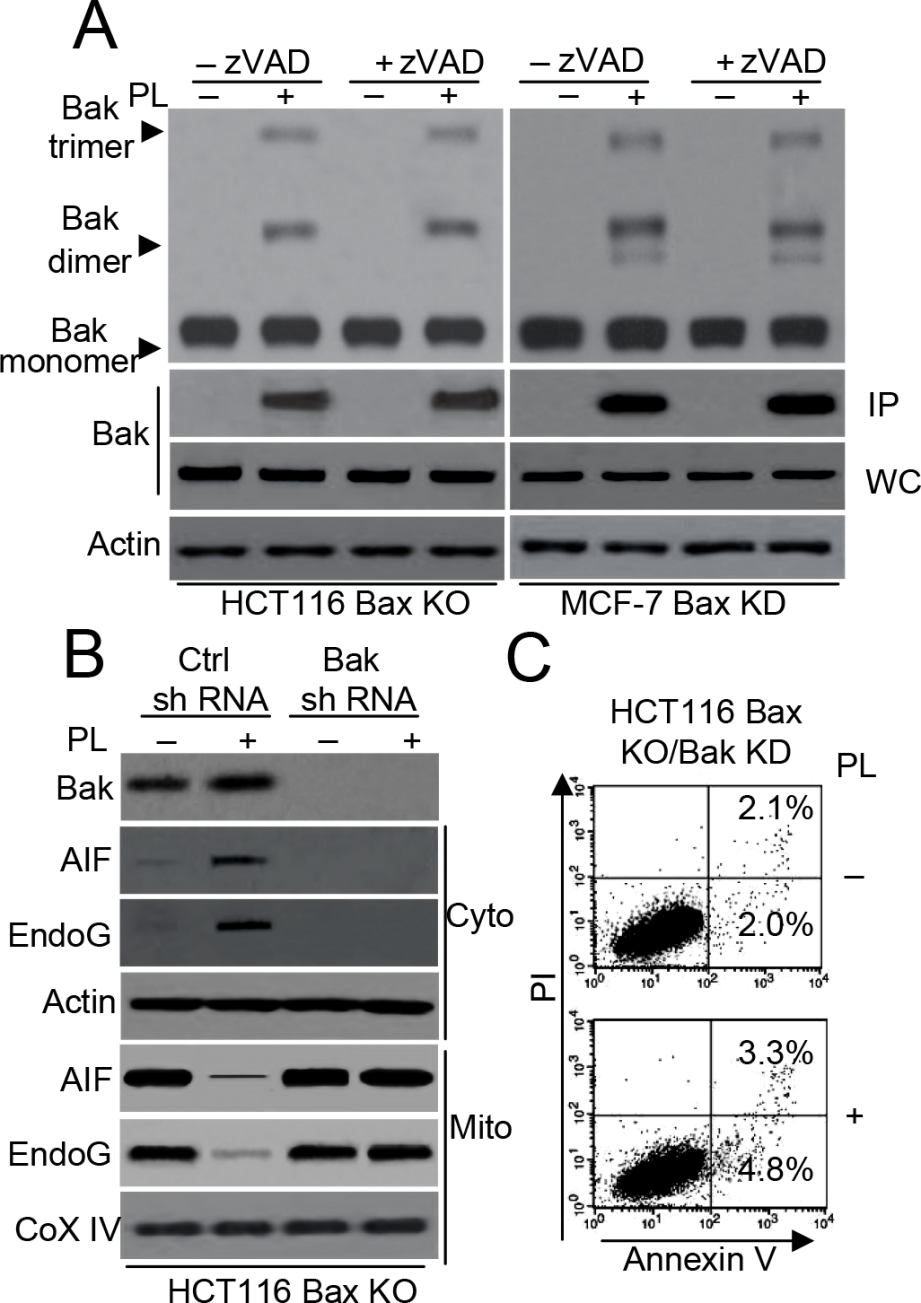
Bak regulates PL-induced cell death in HCT116 Bax KO cells **A.** HCT116 Bax KO and MCF-7 Bax KD cells pretreated with or without 20 μM zVAD for 1 h and then treated with 10 μM PL for 48 h. Bak oligomerization and and conformational change were examined. β-Actin was used as a protein loading control. **B.** HCT116 Bax KO cells were transiently transfected with Ctrl or Bak shRNA vector for 48 h, and then treated with PL (10 μM) for 48 h. Treated cells were subjected to subcellular fractionation. The cytosolic or mitochondrial fractions were immunoblotted. β-Actin and Cox IV was used as a protein loading control. **C.** HCT116 Bax KO cells were stably transfected with Bak shRNA and treated with 10 μM PL for 48 h. Cells were collected for Annexin V/PI staining to detect cell apoptosis. Representative results of three experiments with consistent results are shown.

To further investigate the contribution of Bak to PL-induced cell death, we stably transfected Bax shRNA into MCF-7 cells to knock down Bax expression. Western blot analysis confirmed the lack of Bax and Bak expression in HCT116 Bax KO and MCF-7 Bax KD cells (Supplementary Figure 4E). Our data revealed that PL induces a Bak conformational change and oligomerization in HCT116 Bax KO and MCF-7 Bax KD cells (Figure 4A). Additionally, PL induces Bak oligomerization in MCF-7 cells (Supplementary Figure 4F). We then knocked down Bak expression in HCT116 Bax KO cells. As illustrated in Figure 4B, Bak shRNA effectively decreased Bak expression and the cytosolic release of AIF and endoG after PL treatment. Annexin V/PI staining also demonstrated that cell death is inhibited in HCT116 Bax KO/Bak KD cells following PL treatment (Figure 4C). These results indicate that Bak activation is necessary for PL-induced cell death.

### p53 mediates bak activation in cell death

**Figure 5:**
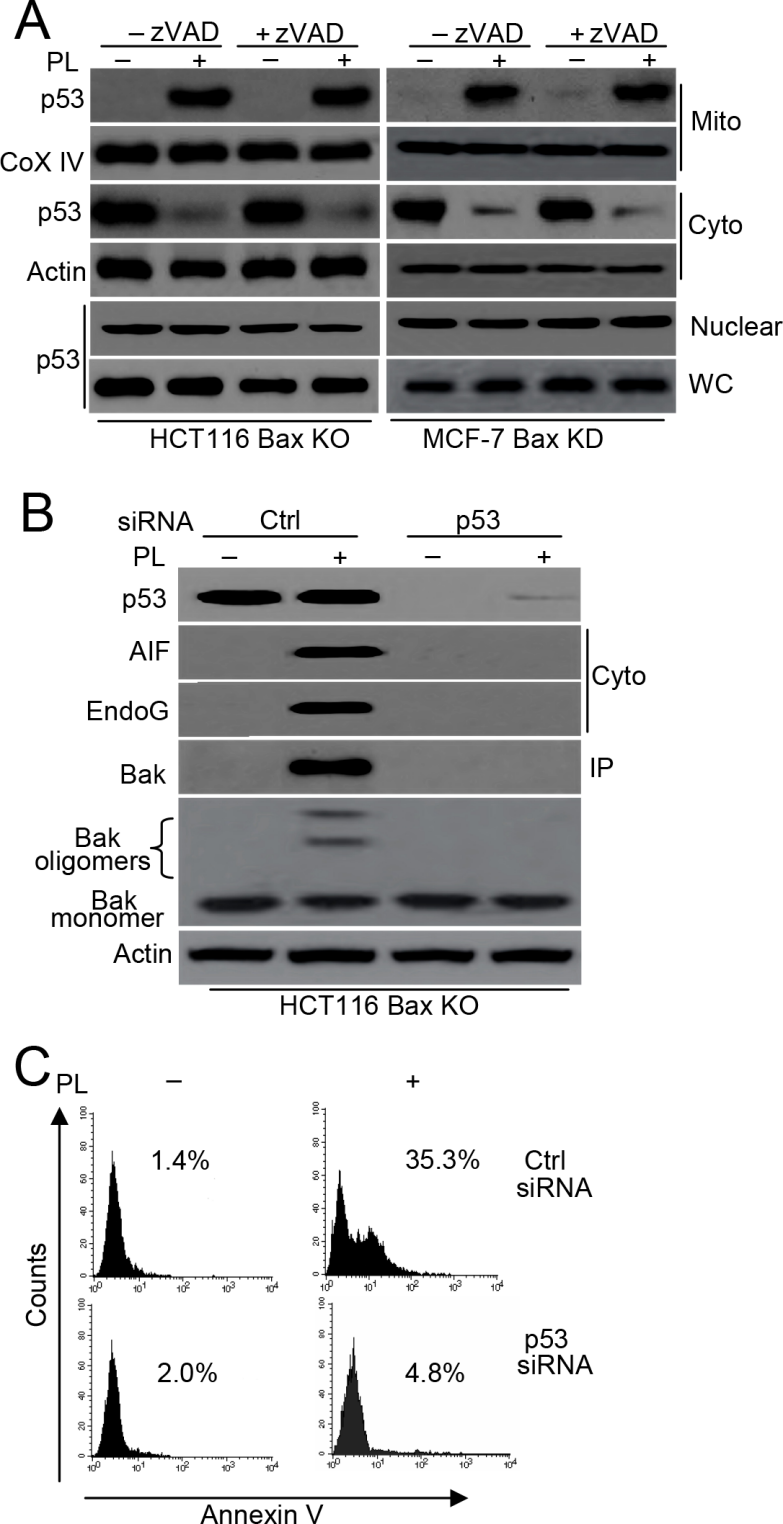
p53 mediates Bak activation **A.** Cells were pretreated with or without zVAD (20 μM) for 1 h, and then treated with 10 μM PL for 48 h. Treated cells were treated subjected to subcellular fractionation. The cytosolic, nuclear or mitochondrial fractions were immunoblotted. β-Actin and Cox IV was used as a protein loading control. WC means whole cell. **B.** Cells were transfected with p53 or Ctrl vector siRNA for 48 h, and then treated with PL for 48 h. one portion of treated cells were subjected to subcellular fraction and detection the release of AIF and endoG. The other portion of treated cells was used to detect Bak conformational change and oligomerization. p53 expression and translocation was examined by p53 antibody. **C.** HCT116 Bax KO cells were transfected with p53 or Ctrl vector siRNA for 48 h, and then treated with PL for 48 h. Cells were collected for Annexin V staining to detect cell apoptosis. All data are representative of three independent experiments.

Both our study and other studies have demonstrated that p53 mitochondrial translocation regulates Bax activation in cell death [3, 9]. Moreover, mitochondrial p53 interacts with Bak to release Cyt c [17]. We therefore wanted to determine whether p53 is involved in Bak-induced cell death following PL treatment. We first detected p53 mitochondrial translocation. Our data confirmed p53 translocation from the cytosol to the mitochondria after PL treatment in HCT116 Bax KO and MCF-7 Bax KD cells (Figure 5A). Our data also demonstrated that PL treatment induces p53 translocation in HCT116 Bax KO, MCF-7 Bax KD and MCF-7 cells in the presence of zVAD (Figure 5A and Supplementary Figure 5A). We then further examined the effects of p53 on Bak activation and cell death. We found that p53 siRNA efficiently prevented AIF and endoG release as well as Bak activation in HCT116 Bax KO and MCF-7 cells (Figure 5B and Supplementary Figure 5B). This decrease in p53 expression also inhibited cell death (Figure 5C and Supplementary Figure 5C).

### Mitochondrial p53 phosphorylation mediates bak activation

**Figure 6:**
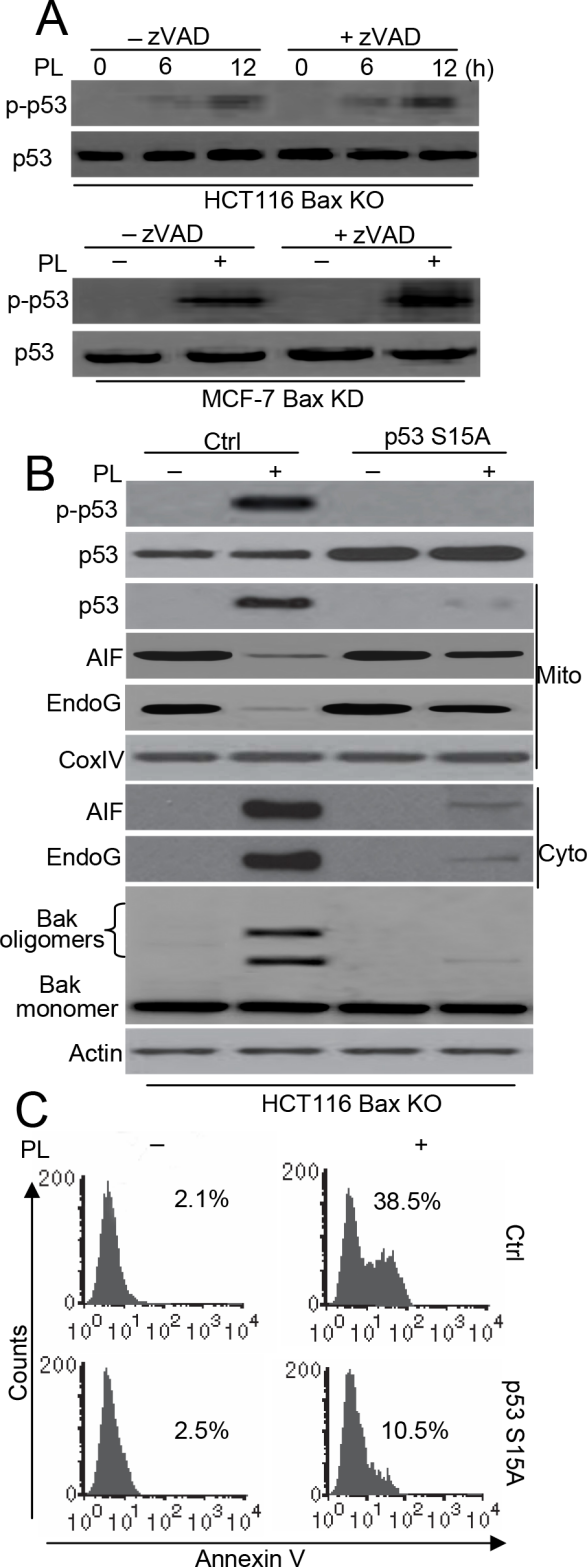
p53 phosphorylation contributes to p53-dependent Bak-induced cell death **A.** Cells were pretreated with zVAD (20 μM) for 1 h, and then treated with 10 μM PL for 48 h. Treated cells were collected for detect p-p53 with specific p53 (Ser15) antibody and p53. **B.** Cells were transfected with Ctrl pCDNA 3.1 or p53 S15A pCDNA 3.1 vector for 48 h, and cells were treated with 10 μM PL for 48 h. Treated cells were collected for detecting Bak oligomerization. Subcellular fractionations were used to detect the release of AIF, endoG. p53 phosphorylation was detected by p53 (Ser 15) antibody. p53 expression and translocation was examined by p53 antibody. β-Actin and Cox IV were used as a protein loading control. **C.** Cells were treated as described in **B** and apoptosis were examined. Cells were stained with Annexin V and detect the Annexin V positive staining using flow cytometry. All data are representative of three independent experiments.

A recent study showed that an increase in p53 mitochondrial content is accompanied by an increase in p53Ser15 phosphorylation in mitochondrial fractions [18]. Moreover, mitochondrial p53Ser15 phosphorylation mediates Bak activation in cell death [19]. We therefore wanted to determine whether the phosphorylation levels of p53 contributed to p53 accumulation and Bak activation in our experiments. We first examined the p53Ser15 phosphorylation. Our experiments showed that p53Ser15 phosphorylation (p-p53) is increased in HCT116 Bax KO cells after PL treatment, regardless of whether there was zVAD pretreatment. Similarly, PL also induces p-p53 in MCF-7 Bax KD and MCF-7 cells (Figure 6A and Supplementary Figure 5A). Moreover, immunofluorescent staining also revealed the accumulation of p-p53 in the mitochondria of HCT116 Bax KO cells (Supplementary Figure 6). To further ascertain the importance of p53Ser15 phosphorylation in cell death, we constructed a pCDNA3.1-p53S15A mutant plasmid and transfected it into cells. We found that our mutant significantly inhibits p53 translocation as well as the release of AIF and endoG. Moreover, Bak activation was prevented by transfection with the p53S15A mutant (Figure 6B). Flow cytometry detection of Annexin V staining confirmed that the p53 mutant inhibits cell death (Figure 6C).

To further understand whether the p53S15A mutant affects endogenous p53 activation, we constructed HA-p53 WT and S15A vectors and transfected them into HCT116 Bax KO cells. We found that a p53 antibody could reliably distinguish exogenous and endogenous p53 expression, as described previously [20] (Supplementary Figure 7A). Our data show that the p53S15A mutant inhibits exogenous and endogenous p53 mitochondrial translocation (Supplementary Figure 7B).

### Akt contributes to mitochondrial p53 phosphorylation and cell death

**Figure 7:**
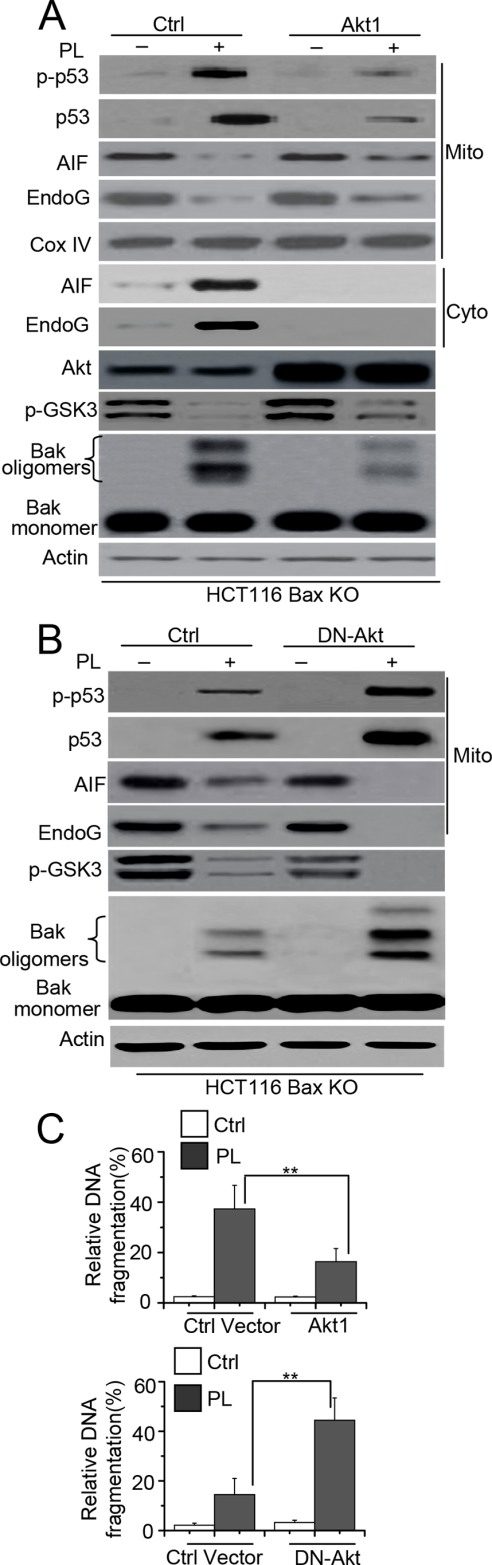
Akt mediates p53 phosphorylation and mitochondrial translocation **A.** HCT116 Bax KO cells were transiently transfected with Ctrl or Akt1 for 48 h, and cells were treated with 10 μM PL for 48 h. One portion of treated cells was subjected to subcellular fraction and detection the release of AIF and endoG. The other portion of treated cells was used to detect Bak conformational change and oligomerization. β-Actin and Cox IV were used as a protein loading control. **B.** Cells were transiently transfected with DN-Akt for 48 h, and then cells were treated with PL for 48 h. treated cells were detection as described in **A. C.** Cells were treated as **A.** or **B.** respectively. Akt1 or ctrl vector transfected cells were treated with 10 μM PL for 48 h. DN-Akt or ctrl vector transfected cells were treated with 10 μM PL for 24 h. Cell death was quantitatively detected by a cell death ELISA kit as described in Materials and methods. Graphs showing results of quantitative analyses (*n* = 3, mean ± S.D. **, *P* < 0.01). Data are representative of at least three independent experiments.

Previous study has demonstrated that Akt contributed to chemoresistance by attenuating the phosphorylation of p53 [21]. Our study also showed that the Akt-p53 pathway mediates Bax activation in cell death. We then determined whether Akt could affect the phosphorylation or mitochondrial translocation of p53 as well as Bak activation. We transfected an Akt1 vector into HCT116 Bax KO or MCF-7 cells and then treated these cells with PL. We found that Akt overexpression increases the phosphorylation of GSK3 (p-GSK3), a downstream target of Akt [22], indicating that Akt signaling activity is enhanced in these cell lines. Our data also show that Akt1 transfection inhibits the phosphorylation or mitochondrial translocation of p53, the subsequent release of AIF and endoG and Bak activation (Figure 7A and Supplementary Figure 5D). To further ascertain whether Akt mediates Bak activation through p53 activation, we transfected DN-Akt into HCT116 Bax KO cells and found that p-GSK3 decreased, which suggests a reduction in Akt signaling activity. Our data show that p53 phosphorylation and accumulation, AIF and endoG release and Bak oligomerization are all increased relative to control cells (Figure 7B). Our experiments also reveal that LY294002, a PI3K/Akt pathway inhibitor, efficiently decreases the phosphorylation levels of Akt (p-Akt). Moreover, p-p53 and p53 accumulation, AIF, endoG release and Bak oligomerization noticeably increases after LY294002 treatment (Supplementary Figure 5E). Cell apoptosis detection by ELISA assay also revealed that the overexpression or inhibition of Akt affects cell death (Figure 7C). These results demonstrate that the Akt pathway mediates cell death following PL treatment.

## DISCUSSION

It is important to isolate natural compounds for the treatment and/or prevention of cancer. A major prerequisite in the use of these agents is that they be physiologically non-toxic and inert towards normal cells. PL is such a compound [23]. PL is a naturally occurring naphthoquinone derivative that has been shown to have antitumor activities against leukemia, breast cancer, and ovarian cancer, among others [23–25], and it has been used in Indian medicine to treat various ailments for more than 2,500 years [24]. There is evidence that the anti-tumor properties of PL are mediated by the inactivation of the Akt/NF-kB signaling pathway and by the inactivation of MMP-9 and VEGF, two proteins that are considered important for the processes of invasion, angiogenesis and metastases [23–26]. Previous studies have confirmed that PL triggers a decrease in the mitochondrial membrane potential and initiates the intrinsic cell death pathway. PL can induce the mitochondrial apoptotic pathway by mediating the activation of Jun N-terminal kinase (JNK) and p53 and the inactivation of Bcl-2 or Bcl-xL [23–25]. Those researchers have investigated the anti-tumor mechanism of PL and indicated that apoptosis plays an important role in the effects of PL treatment. However, the function of PL on cancer chemoresistance is still unknown.

It is well established that some apoptosis-related genes mutate in cancer and can lead to cancer chemoresistance [4, 27]. For example, MCF-7 cells, which lack a functional caspase-3, are chemoresistant to cisplatin [28]. Moreover, caspase-3 deficiency leads to the survival of breast cancer and renders breast cancer cells resistant to chemotherapeutic drug-induced apoptosis [6]. Caspase-8 has been reported to be mutated in colon cancer, and these mutations interfere with apoptosis [29]. Caspase-9 is a potential tumor suppressor in the childhood malignancy neuroblastoma [30]. Recent studies have indicated that caspase-2 acts as a tumor suppressor in Kras-driven lung cancer. Caspase-2 deletion impacts long-term chemotherapy treatment and lung tumorigenesis [31]. These reports revealed that a deficiency in the caspase family of proteins might affect tumorigenesis and cancer therapy outcomes. Thus, it is necessary to find alternative treatments for chemoresistant cancer due to dysfunctional caspases.

Because previous studies have reported that PL could trigger death in MCF-7 cells [23], we speculated that PL could induce caspase-independent cell death in cancer cells. Our experiments confirmed that PL initiated cell death in MCF-7, MCF-7/zVAD, HCT116 and HCT116 Bax KO/zVAD cells. Although PL could induce caspase-3 cleavage in wild type HCT116 cells, the inhibition of caspase activation had little effect on cell death. Our data also revealed that the release of AIF and endoG from mitochondria is an important part of PL-induced cell death. It has been reported that AIF and endoG translocate to the nucleus to trigger caspase-independent cell death [12]. The release of AIF and endoG from mitochondria can induce DNA cleavage and oligonucleosomal DNA breakdown in a sequential fashion [12, 15].

Mitochondria respond to multiple death stimuli, including those involving the pro-apoptotic Bcl-2 family of proteins, such as Bax and Bak, which induce mitochondrial membrane permeabilization and cause the release of apoptotic molecules [9, 14, 32, 33]. Moreover, Bak can induce cell apoptosis in a Bax-dependent or Bax-independent manner [14, 16, 34]. Our experiments demonstrate that Bak mediates Cyt c (data not shown), AIF and endoG release after PL treatment, independent of Bax. Other studies have revealed that the release of AIF and endoG by Bax/Bak is dependent on caspase activation [10]. Some studies have also demonstrated that Bax induces the release of AIF in a caspase-independent cell death pathway [13]. However, our research demonstrated that Bak regulates a caspase-independent cell death pathway through AIF and endoG release. Our study excluded the function of Bax in AIF and endoG release and affirms that Bak can initiate a caspase/Bax-independent cell death pathway.

Both our previous [9] and present study confirm that p53 accumulates in the mitochondria during the process of cell death. Our previous study affirmed the effect of mitochondrial p53 on Bax activation. In the present work, we reveal that mitochondrial p53 can induce Bak activation. Indeed, mitochondrial p53 induces both Bax and Bak activation [17, 35, 36]. Moreover, recent studies have shown that mitochondrial p53Ser15 phosphorylation can mediate Bak activation [19]. Our research also provided the evidence to support this conclusion.

Nieminen and his colleagues demonstrated that the Myc-AMPK pathway mediates p53 phosphorylation and mitochondrial accumulation [19]. However, our research revealed that the Akt pathway mediates changes in p53. These findings highlight certain differences between our study and that by Nieminen and his colleagues. We used cancer cells as cell model, whereas Nieminen and his colleagues used immortalized epithelial cell lines. Furthermore, they utilized Myc-inducible systems, which can enhance Myc expression, to regulate the change of protein activation and cell death. We used PL, a chemical agent, to mediate that alteration. We could not detect a change in Myc expression and AMPK activity (data not shown) in our system. This finding indicates that PL utilizes a separate upstream pathway to regulate p53 and Bak activation. In fact, Fraser and his colleagues revealed that activated Akt attenuated p53 phosphorylation in CP-induced cell death [21]. Our data further promote the findings that Akt mediates p53 phosphorylation and mitochondrial translocation as well as Bak activation. Moreover, the study by Nieminen and his colleagues found that Myc enhances p53 expression in cells, but our data showed that PL has no effect on p53 expression. This result further suggests that we have found a different mechanism underlying the regulation of p53 activation.

It is noteworthy that both our research and the study by Nieminen and his colleagues revealed that p53 had no nuclear accumulation during apoptosis. Nieminen and his colleagues found that active Myc did not lead to an up-regulation of any of the examined transcriptional targets of p53. Additionally, the p53 transactivation-deficient mutant induces Bak activation [19]. We utilized a Bax luciferase plasmid to detect p53 transactivation and found that p53 does not efficiently activate the Bax promoter (Supplementary Figure 8), which suggests that transactivation of p53 does not contribute to PL-induced cell death. Indeed, previous studies have demonstrated that transactivation-deficient mutant p53 can still translocate to mitochondria [37]. Future studies should focus on investigating the detailed mechanism behind p53 activation by upstream factors and subsequent cell death events.

In summary, we elucidate the molecular mechanism of PL action in cancer cells with the absence of caspase activity and provide the first evidence that Bak activation is an important factor for PL-induced cell death with dysfunctional caspase. Furthermore, we demonstrate that Akt negatively regulates p53-mediated Bak activation, independent of caspases (Supplementary Figure 9). A thorough understanding of how PL works in cells may improve the treatment outcomes for human cancer.

## MATERIALS AND METHODS

### Materials

PL, CP, CHX, STS or VP16 were obtained from Sigma (St. Louis, MO, USA). Anti-Fas cross-linking antibody (clone CH11) was obtained from Upstate Biotechnology, Inc. PI and Bak (B5897), and actin (clone AC-74, A5316) antibodies were also from Sigma. Bismaleimidohexane (BMH) was obtaine from Pierce (Rockford, IL, USA). Bak Ab-1 (AM03) was from Merck (Darmstadt, Germany). p53 (clone 7F5, #2527), p53 (Ser 15) (#9284), Akt (#9272), AIF (#4642), CoxIV (#4844) and phospho-GSK-3α/β (Ser21/9) antibodies (#9331) were purchased from Cell Signaling (Beverly, MA). EndoG (ab9647) antibody was from Abcam (Cambridge, UK). Cyt c (sc-13156), Bax N-20 (sc-493) antibodies and (z-Val-Ala-Asp (OMe)-FMK (zVAD)) (sc-311561) were from Santa Cruz (Santa Cruz, CA, USA).

### Gene silencing with small interfering RNAs and plasmids

Small interfering RNA (siRNA) oligonucleotides were purchased from Dharmacon (Lafayette, CO) with sequences targeting Bax (5′- AACUGAUCAGAACCAU CAUGG-3′), Bak (5′-AACCGACGCUAUGACUCAG AG-3′), AIF (5′-GGCUACGUCCAGGAGCGCACC-3′), endoG (5′-AAGAGCCGCGAGUCGUACGUG-3′) and p53 (5′-CGGCAUGAACCGGAGGCCCAU-3′). For Bax or Bak construction, the siRNA was cloned into the pSilencer 2.1-U6 hygro plasmid. pCDNA3.1-Bax plasmid was a gift from Quan Chen (Chinese Academy of Sciences, Beijing, China). Dominant negative (DN) Akt1 was a gift from Dr. Michael J. Quon (University of Maryland at Baltimore, Baltimore, Maryland, USA). The DN-Akt with substitutions of Ala for Lys^179^ in the ATP binding domain, as well as for the regulatory phosphorylation sites, Thr^308^ and Ser^473^, was created and subcloned into pCIS2 as described [3, 38–40]. The constitutively active Akt1 construct HA-PKB-T308D/S473D was obtained as previously described [41, 42]. p53 cDNA was purchased from Origene (Rockville, MD, USA) as previously described [43] and subcloned into pCDNA3.1 vector (Invitrogen, Carlsbad, CA, USA) or pSELECT N-HA-Zeo (Invivogen, San Diego, CA, USA). The p53 S15A mutant was generated by site-directed mutagenesis using Pfu-ultra poly-merase (Stratagen, La Jolla, CA, USA) followed by DpnI digestion (Fermentas Inc., Glen Burnie, MD, USA) according to the manufacturer's instructions.

### Cell culture and transfection

MCF-7, HCT116 cells were obtained from the American Type Culture Collection. MCF-7 cells were maintained at a subconfluent state in RPMI 1640 containing 10% FCS, 200 units/ml penicillin, and 200 μg/ml streptomycin in a humidifiedatmosphere of 5% CO2 at 37°C. HCT116 cells were incubated in DMEM media supplemented with 10% FBS and penicillin-streptomycin. HCT116 Bax KO cells were the gift from Quan Chen (Chinese Academy of Sciences, Beijing, China) [32].

For siRNA or shRNA transfection, cells were seeded on 6-well plates and then transfected with the appropriate plasmid DNA or siRNA using the manufacturers' protocols. Typically, cells were seeded on coverslips in the 6-well plates, and then 1 μg of plasmid DNA or 100 nM siRNA and 4 μl of DMRIE-C reagent (Invitrogen, Carlsbad, CA, USA) were used per coverslip. The cells were incubated for 4 h in the transfection mixture, which was then replaced with fresh culture medium. For stable transfection, cells were transfected with the constructs as previously described [9, 32]. Positive clones were selected with 600 μg/ml hygromycin (Invitrogen) for several weeks.

To establish stable cell lines expressing wild-type Bax, HCT116 Bax-KO cells were transfected with the Bax expression constructs. After transfection, cells were plated out by limiting dilution and selected in the presence of 1 mg/ml of G418 (Invitrogen) for 3 weeks. Individual clones were expanded and cell lysates were harvested and examined for Bax expression by Western blotting as described previously [44].

### Apoptosis assays

Three methods were used to assess PL-induced apoptotic cell death: detection of DNA fragmentation with the Cell Death Detection ELISA kit (Roche Diagnostics), Western blot analysis of Cyt c, AIF or endoG release and measurement of apoptotic cells by flow cytometry (PI staining for sub-G1 or Annexin/PI). The Cell Death Detection ELISA quantified the apoptotic cells by detecting the histone-associated DNA fragments (mono- and oligo-nucleosomes) generated by the apoptotic cells [9].

### Cell fractionation and western blot analysis

Mitochondria, nuclear and cytoplasm from cells were fractionated by differential centrifugation as previously described [9, 45]. Cytosol, mitochondria, nuclear extracts, total lysates and immunoprecipitates were analyzed by Western blot with antibody dilutions as follows: actin at 1:20,000; AIF, endoG, p53, Akt, CoxIV at 1:2,000; and Bax, Bak, Cyt c at 1:1,000.

### Bak or bax oligomerization and bak conformational change

Cells were treated with agents and incubated with 1 mM BMH in 10% DMSO or DMSO alone for 30 min at 25°C. After centrifugation at 5000 g for 25 min at 4°C, the reaction was split into supernatant and pellet fractions. The pelleted material (10 mg total protein) was separated by SDS-PAGE and immunoblotted with anti-Bak antibody to detect Bak or Bax oligomerization [16]. Bak conformational change was performed as described [46]. Briefly, Cells were lysed in 1% CHAPS buffer, and 250 μg of protein was immunoprecipitated using anti-Bak (Ab-1; Merck), which only recognizes Bak that has undergone a conformation change. Immunoprecipitated protein was then subjected to immunoblot analysis by using anti-Bak (Santa Cruz) as primary antibodies.

### Luciferase reporter gene assay

The assay refer to previous study [47]. Briefly, HCT116 cells were transfected with Bax luciferase plasmid (pGL3-Bax-luc, a gift form John C. Reed (Sanford-Burnham Medical Research Institute, La Jolla, CA), pRL-TK, pCDNA empty or pCDNA p53 vector for 48 h. Cells were treated with VP16 or PL for 48 h. Treated cells were lysed and the luciferase activity was measured using a dual-luciferase reporter gene assay system, according to the procedures provided by the manufacturer (Promega, Madison, WI, USA).

### Immunofluorescence staining

The experiments were performed according to our previous report [42]. Cells were seeded in 24-well plates with Lab-Tek Chamber Slides with a Cover (Nalge Nunc International, Naperville, IL) in 500 μl medium and incubated overnight. Cells were then treated with PL for 48 h. Medium was removed and cells were fixed in 4% formaldehyde containing 0.1% glutaraldehyde for 15 min at room temperature (RT). After rinsing with cold PBS (pH 7.4), cells were permeabilized with 0.5% Triton X-100 for 10 min at RT. After blocking with 5% goat serum, Cytochrome c (7H8.2C12, BD Pharmingen, USA), AIF, endoG or p-p53 (1:100 dilution) was added, and the fixed cells were incubated with antibodies at 37°C for 1 h followed by incubation with anti-mouse IgG-FITC (Millipore, 1:128 dilution) for 1 h. After removal of antibodies, cells were rinsed with PBS and mounted with 90% glycerol. Fluorescence was immediately observed using an Olympus DP72 microscope.

### Statistical analysis

Statistical analysis of the differences between the groups was performed using the Student's *t* test with *p* < 0.05 considered statistically significant.

## SUPPLEMENTARY FIGURES



## Abbreviations

PL: Plumbagin
Cyt c: cytochrome c
AIF: apoptosis-inducing factor
endoG: endonuclease G
CP: cisplatin
STS: staurosporine
VP16: etoposide
zVAD: z-Val-Ala-Asp (OMe)-FMK
KO: knockout
KD: knockdown
DN: Dominant negative
p-p53: p53 phosphorylation
p-Akt: Akt phosphorylation
p-GSK3: GSK3 phosphorylation
CHX: cycloheximide
