# Supplementary material for: Mitochondrial p53 phosphorylation induces Bak-mediated and caspase-independent cell death
Source: Oncotarget. 2015 Apr 23;6(19):17192–205. doi: 10.18632/oncotarget.3780 (PMC4627301; doi:10.18632/oncotarget.3780)
Supplement: Supplementary file 1 [file oncotarget-06-17192-s001.pdf]

SUPPLEMENTARY FIGURES

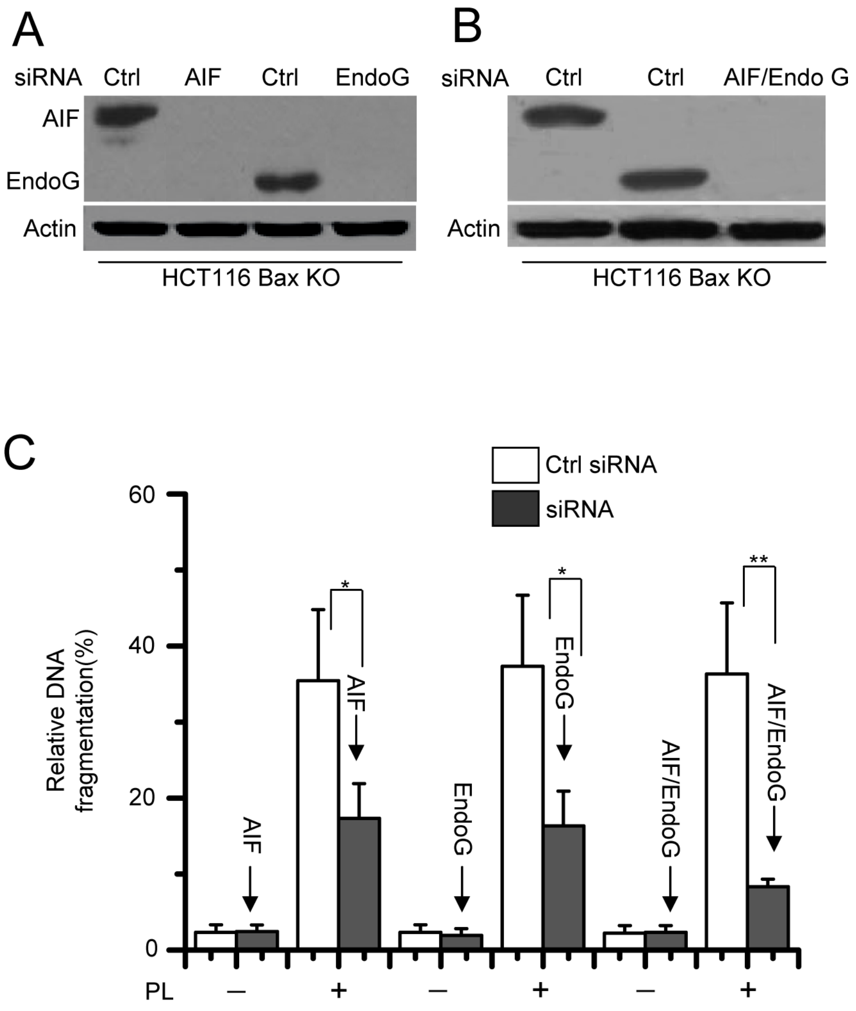

Supplementary Figure 1: The effect of AIF, endoG siRNA on protein expression and cell death. A. HCT16 Bax KO cells were transiently transfected with Ctrl, AIF, or endoG siRNA as described in Materials and Methods. Cells were collected for Western blot analysis. β-Actin was used as a protein loading control. **B.** Cells were transiently transfected with Ctrl or AIF and endoG double siRNA (AIF/endoG) into cells. Cells were collected for Western blot analysis. **C.** cells were treated as described in **A** or **B,** respectively. Cell death was quantitatively detected by a cell death ELISA kit as described in Materials and methods. Graphs showing results of quantitative analyses ($n = 3$, mean ± S.D. *, $P < 0.05$; **, $P < 0.01$). Representative results of three experiments with consistent results are shown.

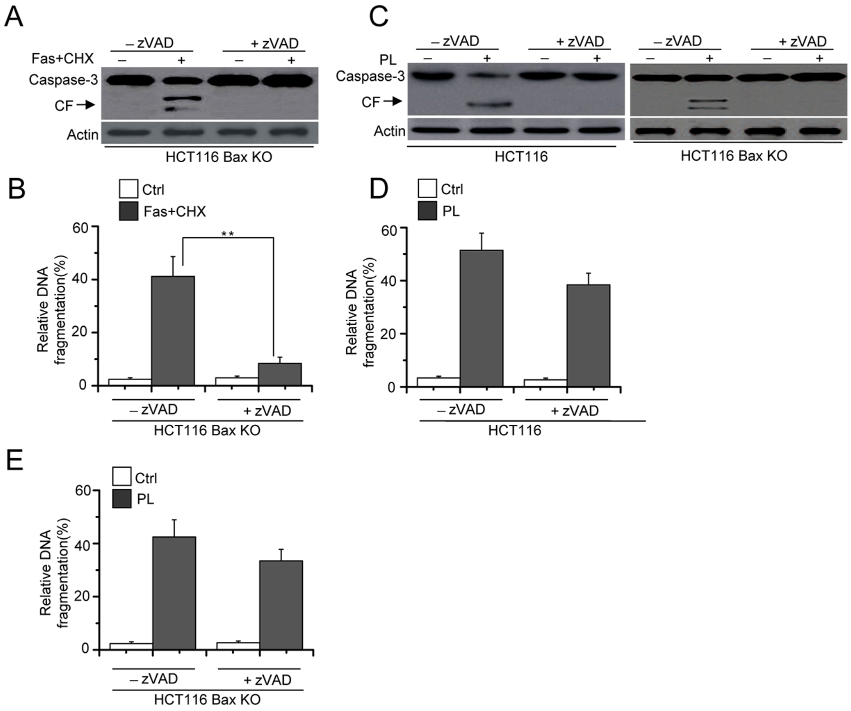

Supplementary Figure 2: The effect of zVAD on caspase-3 cleavage and cell death in HCT116 Bax KO or HCT116 cells. **A.** HCT116 cells were treated with Fas antibody (0.6 μg/ml)/CHX (10 μg/ml) for 12 h with or without zVAD. Treated cells were collected for caspase-3 detection. β-Actin was used as a protein loading control. **B.** Cell death was quantitatively detected by a cell death ELISA kit as described in Materials and methods. Graphs showing results of quantitative analyses (n = 3, mean ± S.D. **, P < 0.01). **C.** HCT116 or HCT116 Bax KO cells were pretreated with or without 20 μM zVAD for 1h and then treated with 10 μM PL for 48 h. Treated cells were collected for caspase-3 detection. β-Actin was used as a protein loading control. **D.** and **E.** Cell death was quantitatively detected by a cell death ELISA kit as described in Materials and methods. Graphs showing results of quantitative analyses (n = 3, mean ± S.D.).

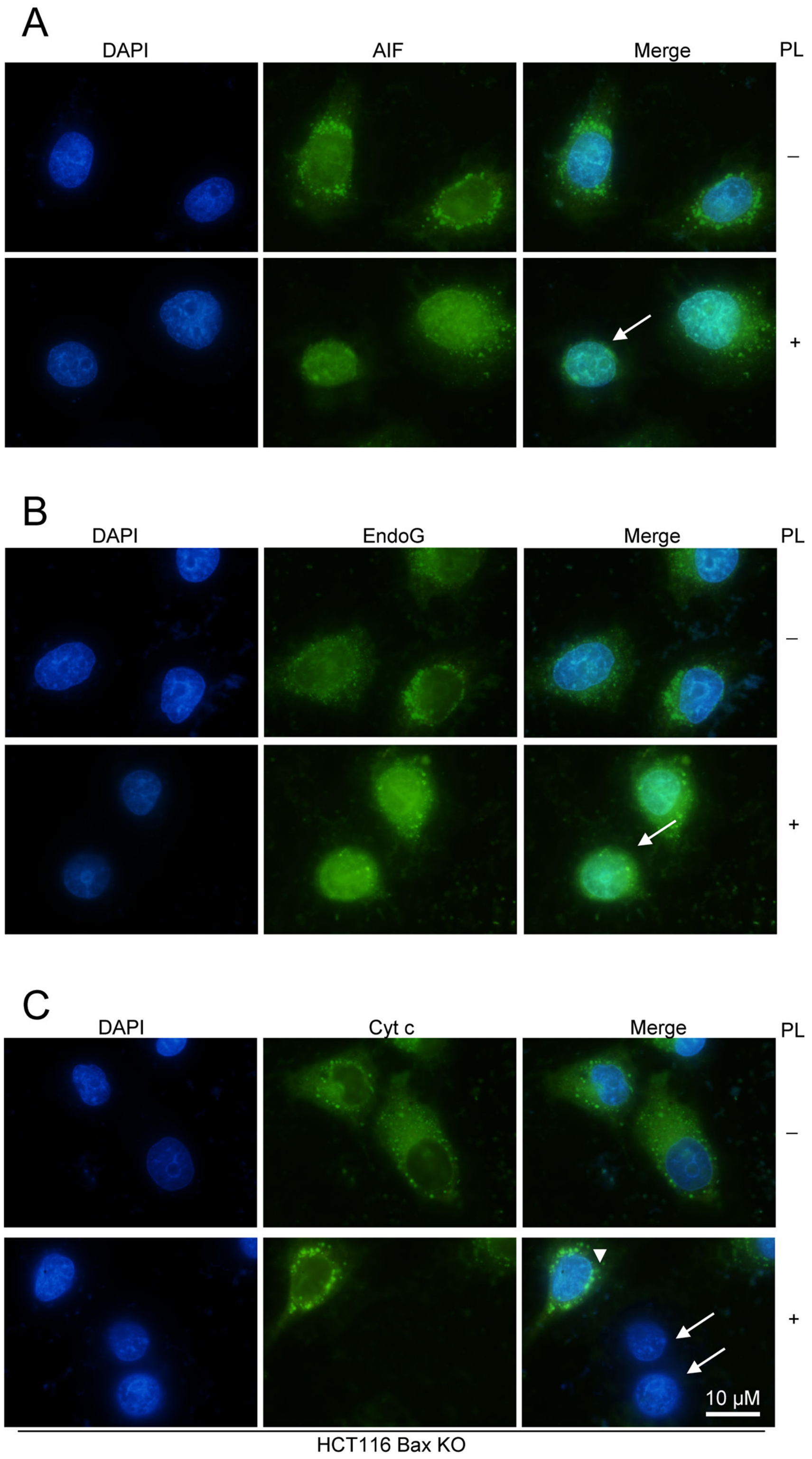

Supplementary Figure 3: The immunofluorescence of AIF, endoG and Cyt c in HCT116 cells. HCT116 cells were treated with PL for 48 h, and then cells were fixed and immunostained for AIF **A.** endoG **B.** or Cyt c **C.** as described in Materials and Methods. Nuclei were counterstained with DAPI. For **A.** and **B.** Arrow indicates the release of AIF or endoG and nuclear translocation. For **C.** Arrowhead indicates the mitochondrial location of Cyt c. Arrow refers to the release of Cyt c. Data are representative of at least three independent experiments.

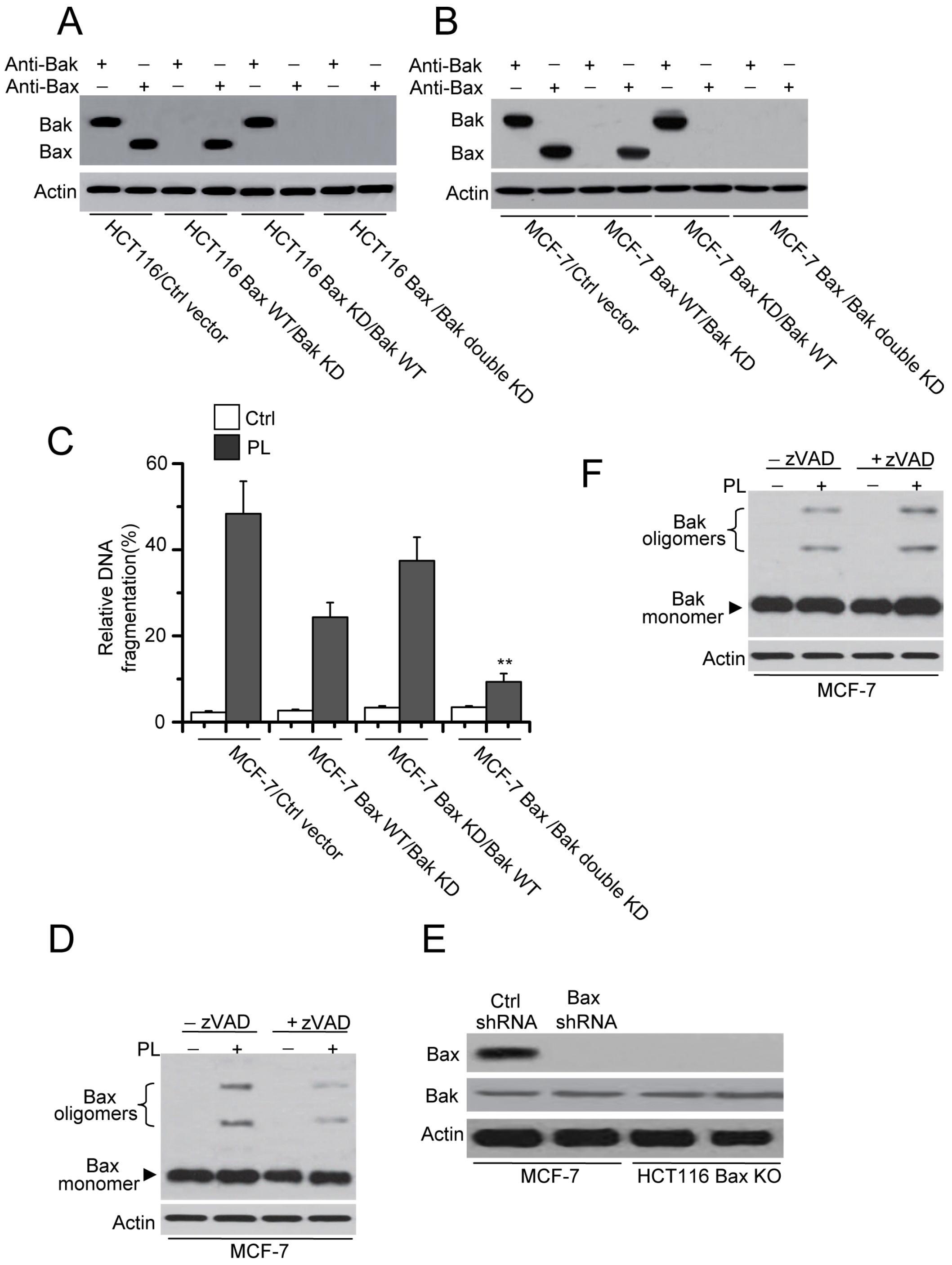

Supplementary Figure 4: The effect of Bax or Bak on cell death. A. HCT116 cells were transfected with Ctrl vector, Bax, Bak or Bax/Bak double shRNA for 48 h. Transfected cells were collected by detection Bax or Bak expression. β-Actin was used as a protein loading control. **B.** MCF-7 cells were transfected with Ctrl vector, Bax, Bak or Bax/Bak double shRNA for 48 h. Transfected cells were collected by detection Bax or Bak expression. **C.** as described in **B.** Transfected cells were treated with PL for 48 h. Treated cells were collected for cell death detection. Cell death was quantitatively detected by a cell death ELISA kit as described in Materials and methods. Graphs showing results of quantitative analyses ($n = 3$, mean ± S.D. **, $P < 0.01$). **D.** MCF-7 cells pretreated with or without 20 μM zVAD for 1 h and then treated with 10 μM PL for 48 h. Treated cells were collected to detect Bax oligomerization. β-Actin was used as a protein loading control. **E.** MCF-7 cells were stably transfected with Ctrl or Bax shRNA vector and cells were collected for detection of Bax or Bak expression. HCT16 Bax KO cells were also collected for detection. β-Actin was used as a protein loading control. **F.** As described in **D** Treated cells were collected to detect Bak oligomerization. β-Actin was used as a protein loading control. Representative results of three experiments with consistent results are shown.

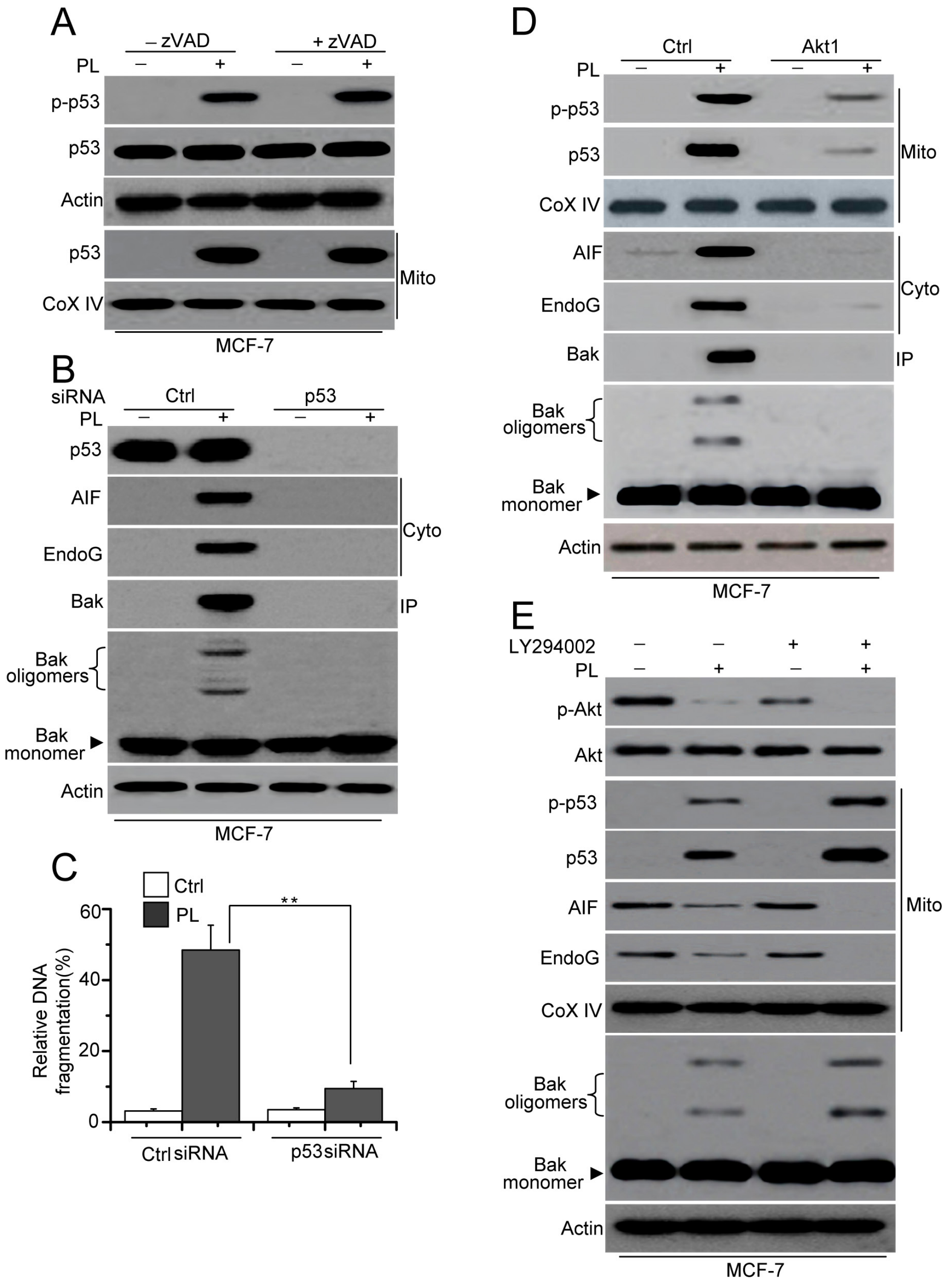

Supplementary Figure 5: The change of p53 and Akt on MCF-7 cells. A. MCF-7 cells pretreated with or without 20 μM zVAD for 1h and then treated with 10 μM PL for 48 h. One portion of treated cells was subjected to subcellular fraction for the mitochondrial of p53 detection. The other of cells was collected to detect p-p53. β-Actin and CoxIV were used as a protein loading control. **B.** MCF-7 cells were transfected with Ctrl or p53 siRNA for 48 h and then treated with 10 μM PL for 48 h. One portion of treated cells was subjected to subcellular fraction for the release of AIF or endoG detection. The other of cells was collected to p53 expression, Bak conformational change and oligomerization. **C.** As described in **B.** Cell death was quantitatively detected by a cell death ELISA kit as described in Materials and methods. Graphs showing results of quantitative analyses ($n = 3$, mean ± S.D. **, $P < 0.01$). **D.** MCF-7 cells were transiently transfected with Ctrl or Akt1 for 48 h, and cells were treated with 10 μM PL for 48 h. One portion of treated cells was subjected to subcellular fraction and detection the release of AIF and endoG. The other portion of treated cells was used to detect Bak conformational change and oligomerization. **E.** MCF-7 cells were treated with 10 μM PL and/or LY294002 (25 μM) for 48 h. One portion of treated cells was subjected to subcellular fraction and detection the release of AIF and endoG, the mitochondrial translocation of p53 and p-p53. The other portion of treated cells was used to detect the change of p-Akt or Akt, Bak conformational change and oligomerization. Data are representative of at least three independent experiments.

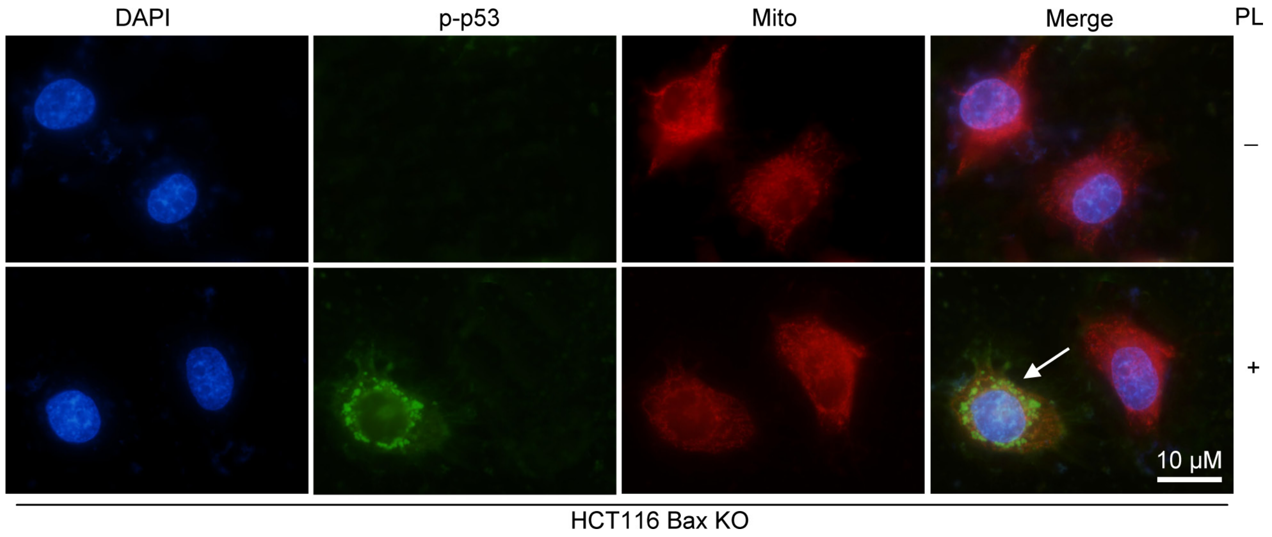

Supplementary Figure 6: The immunofluorescence of p-p53 in cells. HCT116 Bax KO cells were treated with PL for 48 h and cells were collected for detection of p-p53 mitochondrial translocation. The mitochondria were fluorescently stained with 50 nM MitoTracker. Nuclei were counterstained with DAPI. Arrow indicates the mitochondrial translocation of p-p53. Representative results of three experiments with consistent results are shown.

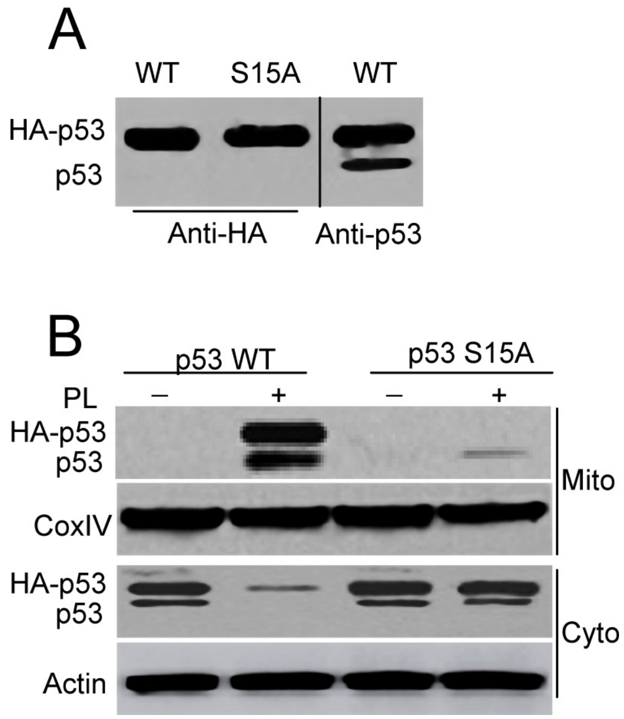

Supplementary Figure 7: The effect of HA-p53 and p53 S15A plasmids on endogenous p53 mitochondrial translocation. A. 100 ng HA-p53 or p53 S15A plasmids were transfected into HCT116 cells, and then cells were collected. Anti-HA and -p53 antibodies detected protein expression. **B.** As described in A, transfected cells were treated by PL. Treated cells were subjected with subcellular fraction and detection for p53 mitochondrial translocation with ani-p53 antibody. β-Actin and Cox IV were used as a protein loading control. All data are representative of three independent experiments.

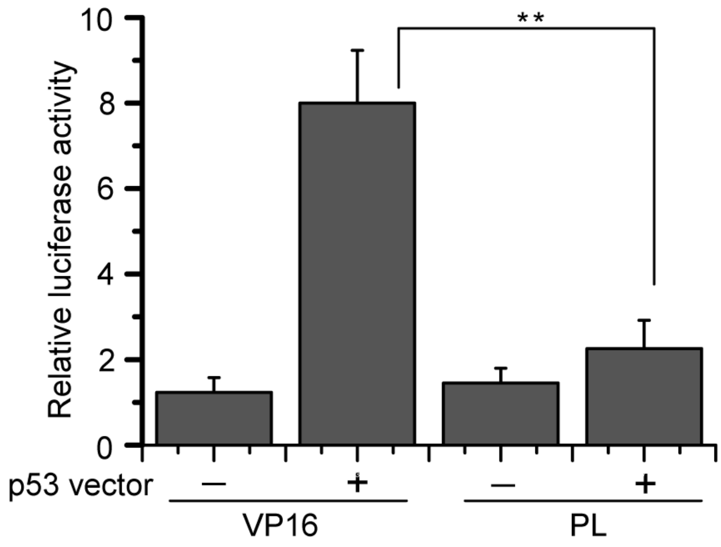

Supplementary Figure 8: The detection of p53 transactivation from Bax promoter in cells. HCT116 Bax KO cells were transfected with 100 ng of Bax luciferase reporter plasmid, 30 ng of second reporter plasmid pRL-TK, 30 ng of p53 plasmid. Approximately 48 h after transfection, the cells were treated by 100 µM VP16 or 10 µM PL for 48 h. Treated cells were lysed and luciferase assays performed. Graphs showing results of quantitative analyses (n = 3, mean ± S.D. **, $P < 0.01$).

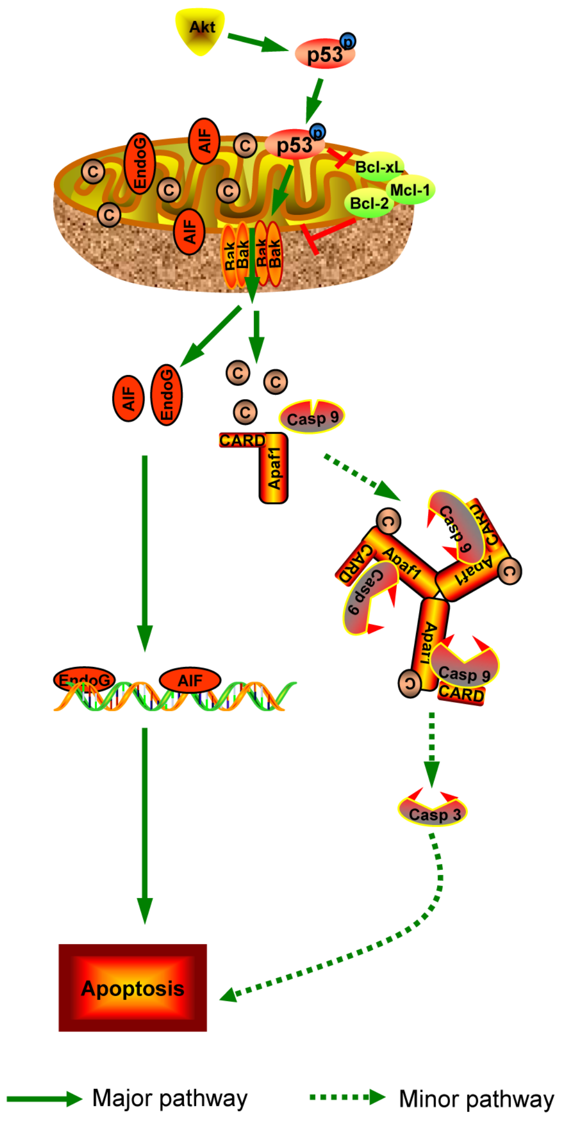

Supplementary Figure 9: A diagram of signaling pathway for PL-induced cell death. PL first decreases the phosphorylation of Akt to inactive Akt. Akt inactivation results in the phoshorylation of p53 and p53 mitochondrial accumulation. p53 translocation induces Bak activation. Activated Bak triggers the release of AIF, endoG, Cytc. In the absence of caspase activation, AIF and endoG can initiate the caspase-independent cell death. It is the major pathway of PL-induced cell death. In the presence of caspase activation, the release of Cyt c can also start caspase-dependent cell death. It is the minor pathway of PL-induced cell death.
